# Editorial: COVID-19 pandemics: Ethical, legal and social issues

**DOI:** 10.3389/fgene.2022.1021865

**Published:** 2022-11-29

**Authors:** Dov Greenbaum, David Gurwitz, Yann Joly

**Affiliations:** ^1^ Department of Molecular Biophysics and Biochemistry, New Haven, NY, United States; ^2^ Reichman University, Herzliya, Israel; ^3^ Zvi Meitar Institute for Legal Implications of Emerging Technologies, Herzliya, Israel; ^4^ Sackler Faculty of Medicine, Sagol School of Neuroscience, Tel Aviv University, Tel Aviv, Israel; ^5^ Department of Human Molecular Genetics and Biochemistry, School of Medicine Sackler Faculty of Medicine Tel Aviv University, Tel Aviv, Israel; ^6^ Centre of Genomics and Policy, Department of Human Genetics, Faculty of Medicine, McGill University, Montreal, QC, Canada

**Keywords:** COVID-19, privacy, pandemic preparedness and response, data sharing, editorial, personal viewpoint, ESLI

## Introduction

Assessing the ethical, legal, and social implications related to the numerous issues that have arisen in the short history of the virus is a hugely valuable effort that was the goal of this special Research Topic, especially as we are likely to experience additional pandemics or pandemic-like medical events in the foreseeable future. Indeed, on 23 July 2022, monkeypox was declared a global health emergency by the World Health Organization (WHO) (Titanji, 2022).

The numerous articles in this Research Topic are testament to the diversity of ethical, legal, and social implications (ELSI) raised by COVID and approaches proposed to address them. They relate to concerns regarding privacy (Beauvais and Maria Knoppers; Song et al.), uncertainty regarding the vaccine (Huang et al.; Kumar et al.; Sun et al.; Zigron et al.), and other prophylactic containment measures and tentative therapeutics (Maaravi et al.), triage guidelines (Merlo et al.) and government guidelines (Fargnoli et al.; Zakar et al.; Zewude et al.), and many more.

Lack of transparency and the lack of trust that this engenders regarding: 1) vaccine approvals by national healthcare authorities; 2) undisclosed contracts between governments and vaccine manufacturers; and 3) prioritization of vulnerable populations, were among the many drivers for vaccine hesitancy or worse in many countries (Qunaibi et al., 2021; Rosenthal and Cummings, 2021; Savoia et al., 2022). As such, among the most effective measures for building public trust in national vaccine drives is assuring clear and detailed transparency on these and other aspects (Cordero, 2021; Gurwitz, 2021; Strully et al., 2021). Although as we have seen with the pandemic and the rise of COVID related conspiracy theories, transparency does not always beget trust; education, access to understandable information, and even good and directed marketing by healthcare stakeholders can all be important components in vaccine trust generation.

However, it is not just past instances relating to COVID vaccination at this intersection of science and society that carry challenging ethical, legal and social implications, but also the permanent or semi-permanent societal and even institutional changes that have been brought on by this pandemic, and that will also create future ELSI. As such, even as most societies have evolved significantly since the first outbreak of this virus, those changes still create issues that ought to be analyzed under the ELSI rubric.

Consider for example the extensive genetic testing infrastructure that has been set up at points of entry around the globe. Most countries have stopped conducting PCR tests on incoming travelers. Eventually all jurisdictions will no longer test for COVID-19 at their borders. At that point, ought we be concerned that this expensive technological infrastructure and their supporting organizational backbones will be repurposed to conduct other forms of genetic testing on incoming foreigners?

This isn’t far-fetched. Recall the shuttle diplomacy that preceded the Russian invasion of Ukraine. The media often presented us with Russian President Vladimir Putin sitting at the head of a comically large table while in discussion with foreign leaders. International reporting confirmed that with foreign leaders unwilling to subject themselves to Russian COVID DNA testing, other appropriate social distancing methods had to be employed, hence the continued use of the long table. (John et al., 2022; Rose, 2022). These leaders were fearful that the Russian COVID testing apparatus was being repurposed such that genetic predispositions to diseases could be uncovered from the DNA of world leaders. (Hessel et al., 2012). Ought we not be concerned that other governments pursue this idea?

With DNA and RNA PCR testing costs continuing to plummet (The Global Genotyping Market size, 2022), it may become feasible for countries to test many of their incoming guests for a host of genetic preconditions scientifically proven, or otherwise, justifiable, or otherwise.

If this is the case, then we should work now to expand genetic privacy protection regimes, even as other areas of privacy are being whittled away (Dobbs, 2022). Thus, we should promote rules and regulations that limit what border officers can and cannot do with genetic information collected to detect an infectious disease, akin to the onerous regulations in place to prevent abuse of data collected through the FBI’s CODIS system that employs genetics to identify individuals in the criminal justice system (34 U.S.C. 407 et seq). Notably, border control authorities in some countries are not forbidden from storing nasal swabs collected for COVID testing for undisclosed future purposes; these biosamples contain, in addition to viral RNAs, nasal epithelial cell DNA of tested individuals. These DNAs may yield tale-tell genetic and epigenomic information on individual lifestyles and disease risks, such as individual DNA methylation profiles (Cardenas et al., 2021).

Our privacy is not only at risk at the borders though. COVID has also brought extensive informative surveillance of wastewater as another tool to assess the level of COVID infections within a given population and the detection of emerging SARS-CoV-2 mutations in the community (Baker and Mallapaty, 2022). And like the potential to find alternative uses for COVID infrastructure, this technology has more recently been used to track Polio outbreaks. However, while the capacity to collect personal identifying information from the thousands of fragments of individual genomes in wastewater has not yet been demonstrated, we can sequence wastewater to track diseases and even drug usage, in particular in isolated communities (Lin et al., 2021). This information could be used to discriminate against historically discriminated minority groups unless we develop rules and regulations to monitor and direct the use of this powerful technology.

However, not all is bad. There are positive externalities resulting from the pandemic. Consider the potential for a drastic evolution in healthcare data sharing and prevention. Heretofore, the human response to such a global peril has been uncoordinated and slow, even as this is clearly not our first rodeo: the first documented human pandemic, the Antonine Plague, dates back almost 2000 years (Cunha et al., 2008).

Since then, medical knowledge and technologies have grown exponentially, and modern communication technologies have revolutionized human relations. Yet, even after two millennia, when COVID-19 struck, the world was again caught unprepared: news of the existence of the disease was slow to circulate; information about the virus, including data on its genetics, evolution, and characteristics of affected patients were unevenly shared (Schriml et al., 2020; Chiara et al., 2021). The results of clinical trials were complicated to obtain (Janiaud et al., 2021). This list of missed opportunities to streamline COVID-19 research, stimulate innovation, and improve our response to the pandemic is incomplete but is sufficient to illustrate our point: with the exception of rapid and open data sharing about newly emerging SARS-CoV-2 strains, the world’s unsatisfactory response to COVID-19, in particular lack of coordination of containment measures on a global scale, is strongly related to a general incapacity to share a broad variety of data covering all aspects of the pandemic as well as research and development in this area.

A chain of important developments that should have prepared us for a public health challenge of this magnitude began unfolding in the middle of the 20th century. The creation of the WHO in 1948 and, the same year, the adoption in the Universal Declaration on Human Rights of Article 27 affirming the right of everyone to enjoy the benefits of scientific progress created both a legal foundation and an international organisation to advocate for health data sharing in a pandemic context (Knoppers et al., 2014). Other important technical, political, and policy accomplishments ensued from these foundational efforts. To name only a few, these include the revision of the WHO International Health Regulations (IHR) to prevent, protect against, control, and provide a public health response to the international spread of disease (2005), the recognition of an international duty to register clinical trials (2008), the implementation of the GISAID database (2008) and the launch of the Public Health Alliance for Genomic Epidemiology (PHA4GE, 2019) (International, 2005; Krleža-Jerić and Lemmens, 2009; Shu and McCauley, 2017; Black et al., 2020). These initiatives and accomplishments are impressive, and viewed together with other similar realisations they would seem to indicate that we now possess both the regulations and infrastructures necessary to enable global data sharing for assuring public health. However, in practice the systematic, rapid implementation of international data sharing by national jurisdictions to help prevent COVID-19 was inconsistent at best (Kalia et al., 2021; Knyazev et al., 2022). Complicating the matter, there has been a lack of consensus over the choice of both data repositories and of the technical standards (Schriml et al., 2020; Griffiths et al., 2022). We believe a way forward is only possible if we can collectively overcome three major hurdles.

## Need to agree on legally binding international regulations for pandemic preparedness

The current WHO system represented by the IHR has proven insufficient to ensure that national governments comply with their international data sharing responsibilities and reporting obligations (Gostin and Katz, 2016). The drafting of a new international instrument was agreed upon at a Special Session of the World Health Assembly that took place in the Spring 2022. Regulations containing pandemic specific extensive, well defined, responsibilities, meaningful sanctions and a transparent reporting system could foster the meaningful collaboration and accountability of state parties. An easier acceptance process makes international regulations’ easier to adopt and more flexible than a treaty in case changes are warranted in the future (Knoppers et al., 2022). Beyond the addressing the responses of national governments, an additional non-binding protocol should address the role and duties of big pharma in times of pandemics. While it would be very challenging and time consuming to convince member states to bind pharmaceutical companies in their territory to specific international legal clauses, instead, using a system of reputational reward the names of companies meeting all requirements of the non-binding protocol, i.e., good corporate citizens, could be displayed on the WHO website. The treaty and protocol should consider the need to promote an interoperable data ecosystem covering all different stages of pandemic evolution, as well as for interim periods between outbreaks. A section of this treaty should provide the accommodations necessary to include lower-middle-income countries (LMIC) and vulnerable population groups as full partners. It goes without saying that representatives from these countries should be given a leading role in determining which accommodation(s) to include here.

## Equity and solidarity

An important failure in data sharing for COVID-19 has been the general incapacity of national jurisdictions to engage LMIC countries and vulnerable population groups through enlisting their participation as active partners in international data sharing efforts (Pratt and Bull, 2021). The reluctance of these stakeholders is perfectly understandable given well-documented past abuses of developing countries and population groups by the research community, corrupt governments and big pharma (Haelewaters et al., 2021). A new arrangement grounded in solidarity and equity is required to address the uneven playing field currently prevailing. For example, as of summer 2022, COVAX has failed to reach its goal of providing COVID vaccine to many developing countries. Future agreements will need to address challenging topics such as benefit sharing for LMIC countries contributing data to research efforts, capacity building, timely access to innovation, intellectual property rights and waivers, additional protection for data from vulnerable minority groups, and a commitment not to present group data in ways that could be conducive to stigmatization and discrimination. A lesson must be learned from situations like that of South Africa who, after sharing information promptly on a new variant, faced stigmatization as a country and at the level of individual residents.

Ultimately concrete solution will need to be implemented to build LMIC countries’ capacity to carry out effective pandemic surveillance and to develop their own data repository. The objective of this strategy is to provide necessary guarantees to obtain access to diverse representative data in terms of gender, ethnic and geographical origin, socio-economic status, etc. So that these data can be used for research to the benefit of the groups having contributed them as well as other populations.

## A change of culture

A determining element of success of the global data sharing strategy we envision here will be the capacity of the WHO and other international organizations, NGOs, and policymakers to propagate an extensive and lasting change of culture towards data sharing. While investigators and data producers in some research fields such as informatics, bioinformatics, and large-scale genomic research have, to a large extent, embraced the open science ethos and are developing incentives and standards to facilitate the process, the same cannot be said of other stakeholders involved in pandemic response. For example, national public health agencies and related departments, pharmaceutical companies, and researchers interested in the socio-economic determinants of health, are less familiar with data sharing requirements, or believe they have little to gain from participating in the process. Proper incentives, beyond the moral duty to contribute to the public good, are necessary to ensure significant buy-in to global data-sharing regulations. Similarly, processes to meaningfully identify and address instances of non-compliance to data sharing policies will need to be devised.

After over 2 years of the COVID-19 pandemic, with populations across the world having felt the negative impacts on population health of pressures on healthcare systems, restrictions on personal freedoms, international travel, and impacts on economic activities, there should be no excuse to further delay the adoption of pandemic data sharing regulations. Such regulations are clearly a necessity to ensure our response to emerging pandemics is well coordinated, meaningful and scientifically optimal. Considerations of equity and solidarity demand that we engage LMIC and other vulnerable groups early in this process to ensure such a framework will address their pandemic needs, not only those of G20 countries. Finally, to be truly effective, the regulations will need to trigger a broad, lasting culture change in favor of rapid data sharing for the benefit of humanity as a whole.

In summary, even as we head into the third year of this pandemic, there remains much to be learned, considered and dealt with, particularly in the areas relating to ethics, law and society. This Research Topic scratched only the surface of the myriad concerns, past, present and future. As we continue facing this global challenge, let us hope that debate and dialogue on these issues will result in policy reforms that will modernise and coordinate the global community capacity to respond to such public health crises in the future. Better harmonization of national public health policies during pandemics will help improve humanity’s preparedness for the forthcoming climate change, already felt in Europe and North America in summer 2022.
